# Interleukin-10 and soil-transmitted helminth infections in Honduran children

**DOI:** 10.1186/s13104-015-1019-x

**Published:** 2015-02-25

**Authors:** Ana Lourdes Sanchez, Dylan Lewis Mahoney, José Antonio Gabrie

**Affiliations:** Department of Health Sciences, Faculty of Applied Health Sciences, Brock University, 500 Glenridge Avenue, St. Catharines, ON Canada L2S 3A1; Department of Biological Sciences, Faculty of Mathematics and Sciences, Brock University, 500 Glenridge Avenue, St. Catharines, ON Canada L2S 3A1; Department of Health Sciences, Faculty of Applied Health Sciences, Brock University, 500 Glenridge Avenue, St. Catharines, ON Canada L2S 3A1

**Keywords:** Soil-transmitted helminths, Geohelminths, Interleukin-10, Th2-type response, Children, Honduras

## Abstract

**Background:**

Soil-transmitted helminths (STH) establish chronic infections in the human intestine. The host reacts to these infections with a dominant T-helper type 2 cell (Th2) response that while attempting to control the worm population, can also provide an anti-inflammatory environment favourable for parasite survival. Regulatory cytokine interleukin 10 (IL-10) has been proposed as a key molecule involved in the attenuation of chronic inflammation and the ensuing tolerance for these helminth parasites. The objective of this study was to determine whether STH-infected children from an endemic community had increased circulating IL-10 levels when compared to non-infected children.

**Results:**

A total of 39 children (25 boys and 14 girls, 7–15 years of age) were enrolled in study. Utilizing the Kato-Katz method to detect intestinal helminthiases, 10 children were non-infected and 29 were harbouring STH infections by *Ascaris lumbricoides*, *Trichuris trichiura* and/or hookworms. Of the 29 infected children, 11 had single-species infections and 18 were polyparasitized with two or three STH species. Serum samples from all 39 children were tested for IL-10 serum concentrations, out of which 12 had undetectable levels while 27 had levels ranging from 0.4-105 pg/mL. Excluding extreme outlying values, 25 samples had IL-10 concentration values ranging from 0.4 -7.2 pg/mL. Differences in IL-10 levels among non-parasitized, monoparasitized, and polyparasitized groups were not statistically significant. However, children infected with any of the three STH species investigated had higher IL-10 levels than non-parasitized children (geometric means: 0.89 pg/mL vs. 0.74 pg/mL, *p* = 0.428). Similarly, polyparasitized children had higher IL-10 levels than both monoparasitized and non-parasitized children (1.04 pg/mL, 0.69 pg/mL, and 0.74 pg/mL, respectively, *p* = 0.481). A significant moderate negative correlation between IL-10 levels and children’s age was found, but no correlations were observed between IL-10 levels and intensity of infection by any of the parasite species investigated.

**Conclusions:**

We found no strong evidence for an association between STH infection and serum IL-10 concentration levels. However, the trends identified here warrant further investigation. Additional research is needed to expand the current understanding of the immune response elicited by STH infections in children living in endemic communities.

## Background

Soil-transmitted helminths (STH) are intestinal parasitic nematodes infecting almost 2 billion people worldwide [1]. The most prevalent STH in the Americas are the common roundworm, *Ascaris lumbricoides*; the whipworm, *Trichuris trichiura*; and the hookworms, *Necator americanus* and *Ancylostoma duodenale* [1]. Although all age-groups are at risk, due to their increased exposure to potentially fecally contaminated soil, children are most commonly infected with STH [2]. Similarly, children tend to harbour most severe infections with resulting detrimental consequences to their growth, nutrition and cognitive function [3,4]. Very importantly, growing evidence indicates that these helminthiases increase susceptibility to other life-threatening infections such as malaria, tuberculosis, and HIV/AIDS [5-7]. In turn, some human studies have shown that intestinal helminths can modulate the occurrence of allergies and asthma [8], alter the course of some autoimmune diseases such as multiple sclerosis [9] and inflammatory bowel disease [10], among others. The latter studies lend renewed support the “hygiene hypothesis” [11,12], more recently called the “Old Friends Hypothesis” [13].

Although knowledge gaps exist regarding the immune mechanisms involved in STH infections [14], it is known that the host-parasite relationship is determined by the interplay of both parasitic manipulation and host tolerance [15] through an efficient induction of a Th2 immune response [16]. The STH-driven Th2-type immunity involves the production of type-2 cytokines, [15,17,18], recruitment of immune cells such as eosinophils, basophils, mast cells, and macrophages, and the differential production of immunoglobulins such as IgG1, IgG4, and notably IgE [18]; elements in common with allergic responses [16]. Because without treatment STH infections are long-lasting and continued re-infection is common, the Th2 polarization is often associated with a regulatory set of cells and cytokines, particularly IL-10 and transforming growth factor β (TGF-β), both of which are significantly linked with hyporesponsiveness and susceptibility to infection [15].

Since its description, IL-10 has been studied for its potent anti-inflammatory properties to prevent excessive immunopathology [15,19,20]. During parasitic infections, as aptly stated by Redpath *et al.,* IL-10 is vital in balancing effective immunity, pathogen persistence, and host pathology [21].

In the field of STH research, this cytokine is subject of increased attention for its potential dual role: dampening inflammation to protect host tissues while inducing tolerance to infection and re-infections [22-26]. The body of literature on the topic is still limited and contains reports of both positive and negative associations between IL-10 and STH infections. Further, the heterogeneity of study designs and methodologies utilized for IL-10 measurement prevents the generation of conclusive evidence either for or against the role of this cytokine in parasitic infection tolerance.

Human studies investigating serum IL-10 levels in STH-parasitized populations are scarce. One of the most recent is a 2012 case–control study nested in an Ecuadorian birth cohort measuring IL-10 plasma levels in 90 children utilizing the same assay reported in the present study. It was found that IL-10 transferred from an STH-infected mother to the unborn child was a predisposing factor for early childhood infection [27]. In 2010, a cross-sectional study of 96 Nigerian schoolchildren with or without *A. lumbricoide*s infection measured IL-10 serum levels by an unspecified enzyme-linked immunosorbent assay (ELISA). This study showed that children with ascariasis had significantly higher IL-10 serum concentrations than non-infected controls [28]. Earlier, in 2006, a study on ascariasis and toxocariasis in India utilized a commercial ELISA to test 46 patients and 19 controls for various cytokines including IL-10. This study found that patients with both infections as well as those with ascariasis only had significantly higher IL-10 levels than healthy controls [29].

Several human *in vitro* studies using either peripheral blood mononuclear cell (PBMC) or whole blood (WB) cultures have also investigated whether IL-10 production was associated with STH infection. Some of these studies measured IL-10 after antigenic stimulation with *T. trichiura* antigens [30] or *A. lumbricoides* extracts [31,32]; whereas others compared spontaneous IL-10 production in non-stimulated cells from infected and non-infected individuals [33,34]. Altogether, these studies report divergent results, ranging from statistically significant associations between IL-10 production and chronic infections [34], intensity of infection [32], environmental conditions [33], to no association at all [30,31].

Arriving at firm conclusions based on research findings such as the ones presented above is somewhat difficult. Some authors propose that measuring cytokine responses in WB cultures is more reflective of *in vivo* conditions than in PBMC cultures [30]. Similarly, cytokine production under controlled conditions might not represent circulating cytokine concentration. Moreover, neither may correspond with the events taking place at the intestinal level where parasites dwell. Clearly, additional research is needed to better understand the role on IL-10 during STH infections.

To obtain grounds for future investigations, the purpose of this study was to determine whether Honduran children infected with soil-transmitted helminths had higher circulating levels of IL-10 compared to non-infected children.

## Methods

### Ethics approval

The present investigation was part of a larger study [35,36] that received clearance from the Research Ethics Boards of Brock University (BU 10–161, 13 January 2011) and the School of Microbiology of the National Autonomous University of Honduras (OF-MEIZ-001-2011, 10 February 2011). Since all participants were minors, both parental informed consent and children’s assent were required prior to enrollment. Parents provided written consents while children’s verbal assents were witnessed by school teachers and recorded on an assent form.

### Study design

This study was designed using a sub-sample of human serum specimens collected from a cross-sectional study done in rural communities of Honduras. The original research study was undertaken in seven rural schools near the city of Catacamas, in the Department of Olancho, about 210 km north-east of Tegucigalpa, Honduras. As previously reported, the majority of children were enrolled in schools providing deworming treatment, either annually or twice a year, but had not received treatment in the past 3 months [35,36].

### Study samples

Of 320 samples comprising the larger study, serum samples from 39 schoolchildren (25 boys and 14 girls, 7–15 years of age) were selected to be tested for IL-10. Samples were chosen to reflect the proportion of parasitism found by means of the Kato-Katz method [1] in the larger sample: 29 (74%) children infected and 10 (26%) non-infected with STH. Of the infected children, 11 and 18 children, respectively, were monoparasitized (single-species helminth infection) or polyparasitized (harbouring more than one STH species). In total, there were 14 *A. lumbricoides* infections (43% light, 50% moderate, and 7% heavy intensity); 26 *T. trichiura* infections (50% light, 38% moderate, and 12% heavy intensity); and 14 with hookworms (100% light-intensity). Intensity of infection had been previously determined based on the number of parasite eggs per gram of feces utilizing the Kato-Katz method [1]. Serum samples had been stored at −20°C since 2011 and had been thawed once before, during shipment.

### Interleukin-10 measurement

Concentration levels of interleukin-10 were measured using a commercial human Interleukin-10 assay (UltraSensitive Sandwich ELISA assay, sensitivity <0.2 pg/mL; Catalog Number KHCO104. Invitrogen, Camarillo, CA, USA). IL-10 ELISA was performed as per manufacturer’s instructions. Eight known standards ranging from 0 pg/mL to 50 pg/mL were used. Samples were ran in duplicates and measured at 450 nm using an automated microplate reader (ELx800, BioTek Instruments, Inc. USA); averaged data was then analyzed using Gen5 Data Analysis Software (BioTek Instruments, Inc. USA).

### Data management and statistical analysis

Infection status was categorized as follows: (i) no infection: no parasites’ eggs observed in the Kato-Katz preparation; (ii) infection with any STH: infection with any of the 3 species under investigation (this includes infections with 1, 2 or 3 species); monoparasitism: infection with a single STH species; and (iv) polyparasitism: infection with 2 or 3 STH species.

IL-10 measurement data were tested for normality and it was found not normally distributed; therefore instead of arithmetic means, geometric means and respective 95% confidence intervals (95% CI) were calculated and used for statistical analyses. To calculate geometric means, IL-10 values below the assay’s detection limit were assigned a value of 0.1 pg/mL. A boxplot analysis was done to detect IL-10 outlying data points (values appearing to deviate markedly from other observations in the study sample). Outlying values, however, were kept in all analyses since non-parametric tests are robust against the presence of such values [37,38]. Correlations among continuous variables (age and infection intensity per species) were explored using Spearman’s rank correlation test. Differences in IL-10 geometric means of non-infected vs. infected children were assessed using the Mann–Whitney *U* test. Differences between non-parasitized, monoparasitized, and polyparasitized groups were tested using the Kruskal-Wallis rank test. These statistical analyses were done using Stata 13 (College Station, TX: StataCorp LP).

## Results

### Interleukin-10 concentrations levels in serum

IL-10 concentration levels and parasitological results from each research participant are presented in Table 1. Of 39 samples, 12 had undetectable levels of IL-10 (0.0 pg/mL). The boxplot analysis (not shown) to detect outlying data points among the 27 samples with measurable IL-10 levels showed four outliers. Two were “mild” outliers (beyond the upper inner fence) and corresponded to non-parasitized children (7.2 pg/mL and 5.1 pg/mL). The other two were “extreme” outliers (beyond the upper outer fence) and corresponded to parasitized children (57.6 pg/mL and 105.0 pg/mL). Excluding the two latter extreme values, 25 samples had concentration levels ranging from 0.4 pg/mL to 7.2 pg/mL. According to the assay’s protocol, expected values for sera range from 1.4 to 8.2 pg/mL (mean 3.6 pg/mL), although the age of tested individuals is unclear [39]. The IL-10 concentration levels expressed as geometric means and respective 95% confidence intervals (95% CI) according to infection status were as follows: (i) non-parasitized children: 0.74 pg/mL (95% CI = 0.26 – 2.09); (ii) parasitized by any STH 0.89 pg/mL (95% CI = 0.42 – 1.88); (iii) monoparasitized: 0.69 pg/mL (95% CI = 0.23 – 2.07); and (iv) polyparasitized: 1.04 pg/mL (95% CI = 0.35 – 3.07).

**Table 1:** **Individual parasitological and immunological results of the study population (n = 39)**

**Participant**	**Age** ^**1**^ **(years)**	***A*** **.** ***lumbricoides*** **EPG** ^**2**^ **(intensity of infection)** ^**3**^	***T. trichiura*** **EPG (intensity of infection)**	**Hookworm EPG (intensity of infection)**	**Number of STH** ^**4**^ **parasite species** ^**5**^	**Interleukin-10 (pg/mL)** ^**6**^
1	≥10	0^5^	0	0	0	0.4
2	≥10	0	0	0	0	1.8
3	≥10	0	0	0	0	0.6
4	≥10	0	0	0	0	7.2
5	≥10	0	0	0	0	0.9
6	<10	0	0	0	0	0
7	<10	0	0	0	0	0.4
8	≥10	0	0	0	0	0
9	≥10	0	0	0	0	1.0
10	<10	0	0	0	0	5.2
11	<10	0	504 (light)	0	1	1.0
12	<10	1008 (light)	0	0	1	1.7
13	≥10	0	960 (light)	0	1	0
14	<10	0	552 (light)	0	1	0
15	≥10	0	0	144 (light)	1	0.7
16	<10	0	168 (light)	0	1	2.1
17	≥10	0	360 (light)	0	1	0
18	<10	0	240 (light)	0	1	2.2
19	≥10	0	0	120 (light)	1	0
20	<10	0	1488 (moderate)	0	1	4.3
21	<10	0	3576 (moderate)	0	1	6.6
22	≥10	0	240 (light)	408 (light)	2	0
23	<10	30624 (moderate)	1704 (moderate)	0	2	0
24	<10	8160 (moderate)	288 (light)	0	2	3.0
25	<10	144 (light)	480 (light)	0	2	57.6
26	<10	20640 (moderate)	12960 (heavy)	0	2	1.5
27	≥10	0	1200 (moderate)	1320 (light)	2	0
28	<10	0	1752 (moderate)	1032 (light)	2	6.4
29	≥10	0	120 (light)	48 (light)	2	1.7
30	≥10	0	10392 (heavy)	936 (light)	2	0
31	<10	336 (light)	1368 (moderate)	0	2	5.2
32	≥10	15264 (moderate)	144 (light)	0	2	0.4
33	<10	12024 (moderate)	2568 (moderate)	360 (light)	3	3.4
34	<10	65880 (heavy)	1320 (moderate)	864 (light)	3	1.6
35	≥10	3912 (light)	2640 (moderate)	144 (light)	3	0.5
36	≥10	1152 (light)	24 (light)	72 (light)	3	105.0
37	≥10	1728 (light)	1704 (moderate)	312 (light)	3	1.2
38	≥10	10800 (moderate)	14400 (heavy)	1320 (light)	3	0
39	<10	7344 (moderate)	24 (light)	48 (light)	3	0

Children were enrolled in primary schools located in seven rural communities of the Department of Olancho, Honduras.

^**1**^Research participants were 7–15 years of age.

^**2**^EPG: eggs per gram of feces.

^**3**^Intensity of infection as determined by the fecal eggs count are classified into light, moderate, or heavy infections as follows, respectively: for *A. lumbricoides*, 1–4,999 EPG, 5,000 – 49,999 EPG and ≥50,000 EPG; for *T. trichiura*, 1–999 EPG, 1,000–9,999 EPG and ≥10,000 EPG; and for hookworms, 1–1,999 EPG, 2,000–3,999 EPG and ≥4,000 EPG [1].

^**4**^STH: soil-transmitted helminth.

^**5**^A value of 0 indicates that no parasite eggs were observed in the microscopic analysis.

^**6**^A value of 0 pg/mL indicates that the sample contained undetectable (<0.2 pg/mL) levels of IL-10.

No association was found between IL-10 levels and infection by individual species (*A. lumbricoides*, *p* = 0.285; *T. trichiura*, *p* = 0.650; hookworms, *p* = 0.357).

**Figure 1:**
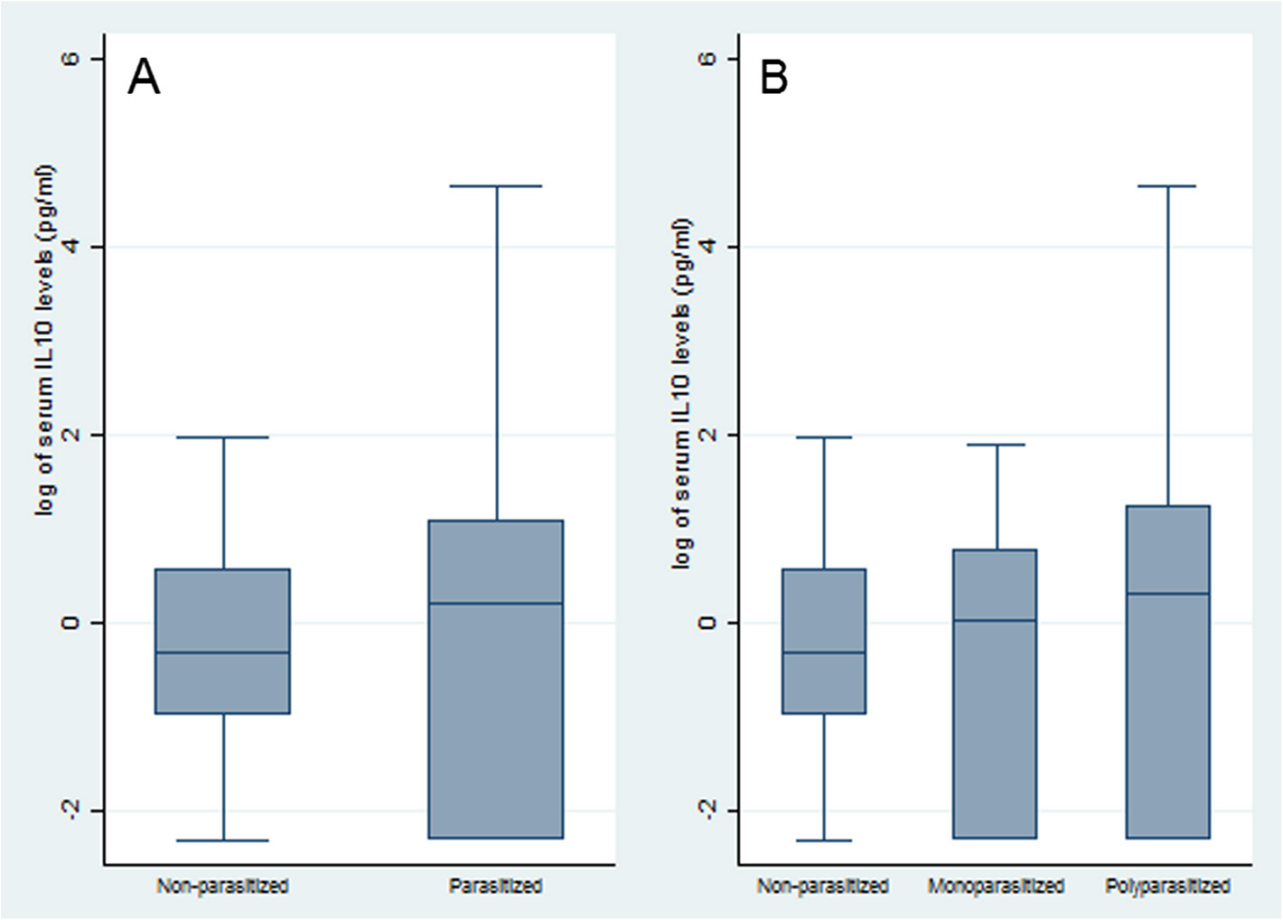
**Differences in IL-10 serum levels according to infection status of the study population (** ***n*** **= 39).** Children were enrolled in primary schools located in rural seven communities of the Department of Olancho, Honduras. **A)** Comparison of serum IL-10 levels in non-parasitized children and children parasitized with one or more species of soil-transmitted helminths as detected with the Kato-Katz method (*Ascaris lumbricoides*, *Trichuris trichiura*, and hookworms). Children infected with any of the three STH species investigated had higher IL-10 levels than non-parasitized children (geometric means: 0.89 pg/mL vs. 0.74 pg/mL *p* = 0.428). Statistical comparisons were conducted using Mann–Whitney *U* test (Wilcoxon rank-sum). **B)** Comparison of serum IL-10 levels in non-parasitized, monoparasitized (infection with a single species) and polyparasitized children (infection with 2 or more species). Polyparasitized children had higher IL-10 levels than both non-parasitized and monoparasitized children (1.04 pg/mL, 0.74 pg/mL and 0.69 pg/mL, respectively, *p* = 0.481). Statistical comparisons were conducted using Kruskal-Wallis rank test. Boxplots show median values (horizontal center line), inter-quartile range (box margins), and 95% confidence intervals (bars).

Differences in IL-10 levels among infection status groups were not statistically significant. However, as shown in Figure 1, as the number of species parasitizing increased, IL-10 levels tended to increase: children infected with any of the three STH species investigated had higher IL-10 levels than non-parasitized children (geometric means: 0.89 pg/mL vs. 0.74 pg/mL, *p* = 0.428) (Figure 1A). Similarly, polyparasitized children had higher IL-10 levels than both non-parasitized and monoparasitized children (1.04 pg/mL, 0.74 pg/mL and 0.69 pg/mL, respectively, *p* = 0.481) (Figure 1B).

Spearman’s rank correlation coefficients showed a significant moderate negative correlation between IL-10 levels and age (*r*_*s*_ = −0.4839, *p* =0.002). No correlations were found between IL-10 levels and intensity of infection (*A. lumbricoides*, *p* = 0.585; *T. trichiura*, *p* = 0.793; hookworms, *p* = 0.289).

## Discussion

Growing research evidence underscores the paramount importance of investigating the role of IL-10 during soil-transmitted helminthiases as it may increase susceptibility for these infections [27]. Further, it has been argued that large-scale deworming programs as currently implemented by recommendation of the World Health Organization [1], will have an impact on the Th1/Th2 immune profile of target populations, which may increase the risk for inflammatory diseases and allergies [40].

The present study is, to our knowledge, the first examining an association between STH infections and IL-10, not only in Honduras but in the Central American region, an area endemic for many parasitic infections including STH [41].

Our data reveals some interesting points. First of all, we detected a wide range of serum IL-10 concentrations among the studied children, from 0 pg/mL (non-detectable) to 105 pg/mL. Similarly, a study by Malla *et al*. in India reported ranges from 0 to 60 pg/mL in infected patients and from 0 to 5 in the control groups [29]. In the Ecuadorian study, IL-10 mean concentrations in cord blood ranged from 0 to 5 pg/mL, with a few outlying values of < 20 pg/mL [27]. In contrast, a study in Nigeria found much higher serum concentrations in children 6–10 years of age (152.5 ± 93.6 pg/mL) or older (177.5 ± 51.9 pg/mL) [28]. Considering that there is no expert agreement on what the healthy values for serum or plasma IL-10 are in the human population [42], the disparity of data suggests that each study must attain its own interpretation depending on their comparison group. In our view, this highlights the need for researchers to present a detailed methodological approach so the knowledge generated can be analyzed and compared appropriately.

We also found that serum IL-10 levels and age were negatively correlated; *i.e.*, as children’s age increased, IL-10 levels tended to decrease. This finding seems unexpected for an endemic country where STH exposure -and therefore immune tolerance to helminths- is likely to increase with age. However, in populations where deworming treatment is offered through school-based programs, the natural patterns of infection are altered. It is worth mentioning that in the larger study originating the present investigation, age was not found significantly associated with STH infection. However, we observed that a one year increment in children’s age reduced their odds for ascariasis by 20% and increased their odds for *T. trichiura* and hookworm infections by 15% and 20%, respectively [36]. We proposed that such variations might have been linked to children receiving annual single-dose albendazole treatment, as this regimen is more efficacious for *A. lumbricoides* than for the other two species [43]. The inverse association between IL-10 and age reported in this study might also be confounded by deworming treatment. Albendazole shortens the duration of infections and/or reduces worm burden, depending on the infecting species [44]. Repeated albendazole treatments have a significant effect in the way children respond to immune stimulus [45], and an immune modulatory effect by IL-10 is likely more evident in chronic or high-intensity infections [46]. In fact, significant differences between chronic STH infections and elevated levels of IL-10 were observed in a study conducted in Ecuador [34]. We were not able to ascertain chronicity of infection in the present study, so this question remains an interesting challenge for future research.

In line with our findings, a study investigating IL-10 serum levels in Nigerian schoolchildren also found that age and intensity of *A. lumbricoides* infection were inverse correlated with IL-10, but a potential explanation for this correlation was not proposed by the authors [28]. Some *in vitro* studies have too explored this association. In a cross-sectional study in Cameroonian children in which a negative correlation between *T. trichiura*-specific IL-10 production and age was found; the authors concluded that IL-10 regulatory effects might be more likely in younger persons [30]. Nevertheless, observations from cross-sectional studies are unlikely to clarify this issue. Empirical evidence from longitudinal studies would help elucidating the effects of deworming and age on children’s immune response to parasitic infections.

No evidence for an association was found between parasitism by individual STH species and IL-10 levels. That these findings are in concordance with some studies [30,31] but in disagreement with others [28,29] is not entirely surprising. In the particular case of *A. lumbricoides* infections, contrasting results are reported throughout the literature [47,48].

In terms of mono- and polyparasitism, we observed, in congruence with other studies [27,28], that as the number of species parasitizing increased, IL-10 levels tended to increase. We could speculate that once parasitized with one species, children who build tolerance through higher levels of IL-10 could be more susceptible to infection with another species. Notwithstanding, it is necessary to be cautious when interpreting data from populations where polyparasitism is common, as very little is known about the immune interactions taking place in a host harbouring multiple parasite species. Moreover, as recently proposed, helminths may steer the host’s immune system not just directly but also indirectly by influencing the composition of the gut microbiome, which in turn may participate in the modulation of inflammatory processes [49].

We also investigated a potential association between intensity of infection (worm burden) and IL-10 levels, but none was found. Several reasons may explain this finding. Although Kato-Katz is the standard method for categorizing infection intensity [1], its sensitivity for a single-stool sample examination varies according to the STH species: >90% for both *A. lumbricoides* and *T. trichiura*, but <70% for hookworm infections [50]. Also, the number of eggs laid by female worms is subject to great variations [51].These two aspects suggest that misclassification of infection cannot be ruled out. In addition, variations in IL-10 levels may be caused not only by ongoing or past infections [21,24], but by host-related factors as well [52]. Undoubtedly, IL-10 regulation is extremely complex and many questions are yet to be answered [24].

In sum, given that IL-10 production and its modulatory effects during STH infection are likely determined by a combination of factors [30,45,53], it is crucial that future studies collect precise information on the deworming history of research participants, and that efforts are made to obtain accurate estimations of worm burden and length of infections. As well, future investigations should strive to analyze research participants’ socio-economic and environmental data as there is evidence that a number of environmental exposures [33] including the presence of a household latrine [52] have a modulatory effect on serum IL-10 levels. Furthermore, emerging evidence suggests that there is significant genetic component to susceptibility or resistance to STH infection [52], and that there may IL-10 genetic polymorphisms that protect against helminthic infections [54].

It is important to acknowledge the limitations of the present investigation. Firstly, we tested a small sample size from a cross-sectional study, which limits our ability to reach firm conclusions. Secondly, other parasitic infections that may shift the Th1/Th2 balance (*e.g.,* with intestinal protozoa or other helminths such *Strongyloides stercoralis*) were not assessed. Thirdly, a detailed medical history was not obtained from the participating children, thus other condition causing increased IL-10 levels such as asthma [55], bacterial infections [56,57], viral infections, and autoimmune diseases [58], cannot be excluded. Finally, a complete immune profile of the studied children (including IL-10 to IFN-γ ratio) was not determined. We recommend future studies accounting for these limitations.

## Conclusion

We provide for the first time Honduran data on childhood STH infections and their association with serum IL-10 levels. Given the small sample size, our analysis was exploratory but a positive trend was identified between the number of species parasitizing and IL-10 levels. Our results highlight the need for further research investigating the immune response elicited by STH infections in children living in endemic areas.
